# *In Vitro* and *In Vivo* High-Throughput Assays for the Testing of Anti-*Trypanosoma cruzi* Compounds

**DOI:** 10.1371/journal.pntd.0000740

**Published:** 2010-07-13

**Authors:** Adriana M. C. Canavaci, Juan M. Bustamante, Angel M. Padilla, Cecilia M. Perez Brandan, Laura J. Simpson, Dan Xu, Courtney L. Boehlke, Rick L. Tarleton

**Affiliations:** 1 Center for Tropical and Emerging Global Diseases, University of Georgia, Athens, Georgia, United States of America; 2 Instituto de Patología Experimental - CONICET, Universidad Nacional de Salta, Salta, Argentina; 3 Department of Cellular Biology, University of Georgia, Athens, Georgia, United States of America; McGill University, Canada

## Abstract

**Background:**

The two available drugs for treatment of *T. cruzi* infection, nifurtimox and benznidazole (BZ), have potential toxic side effects and variable efficacy, contributing to their low rate of use. With scant economic resources available for antiparasitic drug discovery and development, inexpensive, high-throughput and *in vivo* assays to screen potential new drugs and existing compound libraries are essential.

**Methods:**

In this work, we describe the development and validation of improved methods to test anti-*T. cruzi* compounds *in vitro* and *in vivo* using parasite lines expressing the firefly luciferase (luc) or the tandem tomato fluorescent protein (tdTomato). For *in vitro* assays, the change in fluorescence intensity of tdTomato-expressing lines was measured as an indicator of parasite replication daily for 4 days and this method was used to identify compounds with IC_50_ lower than that of BZ.

**Findings:**

This method was highly reproducible and had the added advantage of requiring relatively low numbers of parasites and no additional indicator reagents, enzymatic post-processes or laborious visual counting. *In vivo*, mice were infected in the footpads with fluorescent or bioluminescent parasites and the signal intensity was measured as a surrogate of parasite load at the site of infection before and after initiation of drug treatment. Importantly, the efficacy of various drugs as determined in this short-term (<2 weeks) assay mirrored that of a 40 day treatment course.

**Conclusion:**

These methods should make feasible broader and higher-throughput screening programs needed to identify potential new drugs for the treatment of *T. cruzi* infection and for their rapid validation *in vivo*.

## Author Summary

The treatment of *Trypanosoma cruzi* infection (the cause of human Chagas disease) remains a significant challenge. Only two drugs, both with substantial toxicity, are available and the efficacy of these dugs is often questioned – in many cases due to the limitations of the methods for assessing efficacy rather than to true lack of efficacy. For these reasons relatively few individuals infected with *T. cruzi* actually have their infections treated. In this study, we report on innovative methods that will facilitate the discovery of new compounds for the treatment of *T. cruzi* infection and Chagas disease. Utilizing fluorescent and bioluminescent parasite lines, we have developed in vitro tests that are reproducible and facile and can be scaled for high-throughput screening of large compound libraries. We also validate an *in vivo* screening test that monitors parasite replication at the site of infection and determines the effectiveness of drug treatment in less than two weeks. More importantly, results in this rapid in vivo test show strong correlations with those obtained in long-term (e.g. 40 day or more) treatment assays. The results of this study remove one of the obstacles for identification of effective and safe compounds to treat Chagas disease.

## Introduction

Chagas disease, caused by the protozoan parasite *Trypanosoma cruzi*, is the leading cause of cardiac disease in many countries of Latin America. The World Health Organization has estimated that 16–18 million people are currently infected and 90 million are at risk of acquiring the infection [1]–[2]. Both of the available compounds for treatment, benznidazole (Radanil®, Roche, Rio de Janeiro) and nifurtimox (Lampit®, Bayer, Leverkusen), have potential side effects, require long courses of treatment, and exhibit variable efficacy [3]–[6]. Therefore, there is an urgent need to discover new treatment options with reduced toxicity. Several compounds have been proposed as new therapies for *T. cruzi* infection, however, few have moved beyond the candidate stage.

The development of *in vitro* and *in vivo* high-throughput assays for the screening of anti-*T. cruzi* compounds is essential. Epimastigotes of *T. cruzi* can be easily obtained in abundance from axenic culture and drug efficacy determined using a variety of approaches, including manual, spectrophotometric [7]–[11] or fluorometric [12]–[13] assessment of parasite growth. In addition to the fact that these epimastigote-based assays may not truly reflect the effectiveness of compounds on the life cycle stages of *T. cruzi* that are present in mammals (the extracellular trypomastigotes and intracellular amastigotes) [14], these assays may be laborious and difficult to scale up for high-throughput screening (HTS) [15]–[16]. Assays to detect drug susceptibility of the more appropriate *T. cruzi* life cycle stage, the intracellular amastigotes, have been modified to use parasites expressing the *Escherichia coli* β-galactosidase gene (*lacZ*) [15] and this assay has also recently been scaled up for HTS [17]. However one downside of this method is that it is a single endpoint assay requiring post-assay processing to interpret.

With respect to in vivo drug testing of anti-*T. cruzi* compounds, the vast majority of studies use a mouse model system where the compounds are administered early in the acute phase of the infection [18]–[20], with the main criteria of treatment efficacy based on the suppression of acute parasitemias, the measurement of mortality rates post infection, and/or on the use of parasite detection techniques that frequently yield negative results (i.e. fail to detecte parasies) even in the absence of treatment [6], [18], [20]–[22].

In this study we describe the generation of *T. cruzi* parasite lines that express either the firefly luciferase (luc) or the tandem tomato fluorescent protein (tdTomato) and the use of these lines to establish accurate and simple *in vitro* as well as *in vivo* systems to screen and test anti-*T. cruzi* compounds. tdTomato red fluorescent parasites were detectable by microscopy and flow cytometry and their fluorescence intensity was easily quantified using a fluorescence plate reader. Moreover, the replication of epimastigotes or amastigotes could be monitored at multiple time points over the culture period rather than at endpoint, providing a more accurate assessment of parasite growth kinetics. Bioluminescent as well as fluorescent parasites were detectable via *in vivo* imaging after infection in mice and their expansion was used to rapidly assess the *in vivo* efficacy of anti-*T.cruzi* compounds. These results highlight the use of the methods described here as powerful new tools for the more rapid and efficient high-throughput screening of potential trypanocidal drugs *in vitro* and *in vivo*.

## Methods

### Ethics Statement

All animal protocols were approved by the University of Georgia Institutional Animal Care and Use Committee.

### Parasites and culture procedures

Epimastigote forms of *T. cruzi* CL WT or tdTomato strain were cultured at 27°C in supplemented liver digest-neutralized tryptose (LDNT) medium as described previously [23]. After 3–4 days in LDNT, epimastigotes were submitted to a stress in triatome artificial urine (TAU) medium for 2 h [24]. Then, parasites were incubated in TAU3AAG medium [24] for 6–7 days, at the end of which the highest number of metacyclic trypomastigote was generally obtained. Parasites were then incubated in complement-active FBS to lyse the remaining epimastigotes (by complement activity). Vero cell monolayer cultures were infected with metacyclics to generate trypomastigotes to use in the amastigote growth inhibition assays. Infected Vero cells were cultured in RPMI 1640 medium with 10% fetal bovine serum (FBS) in a humid atmosphere containing 5% CO_2_ at 37°C.

### Generation of fluorescent (tdTomato) *Trypanosoma cruzi*

**Figure 1:**
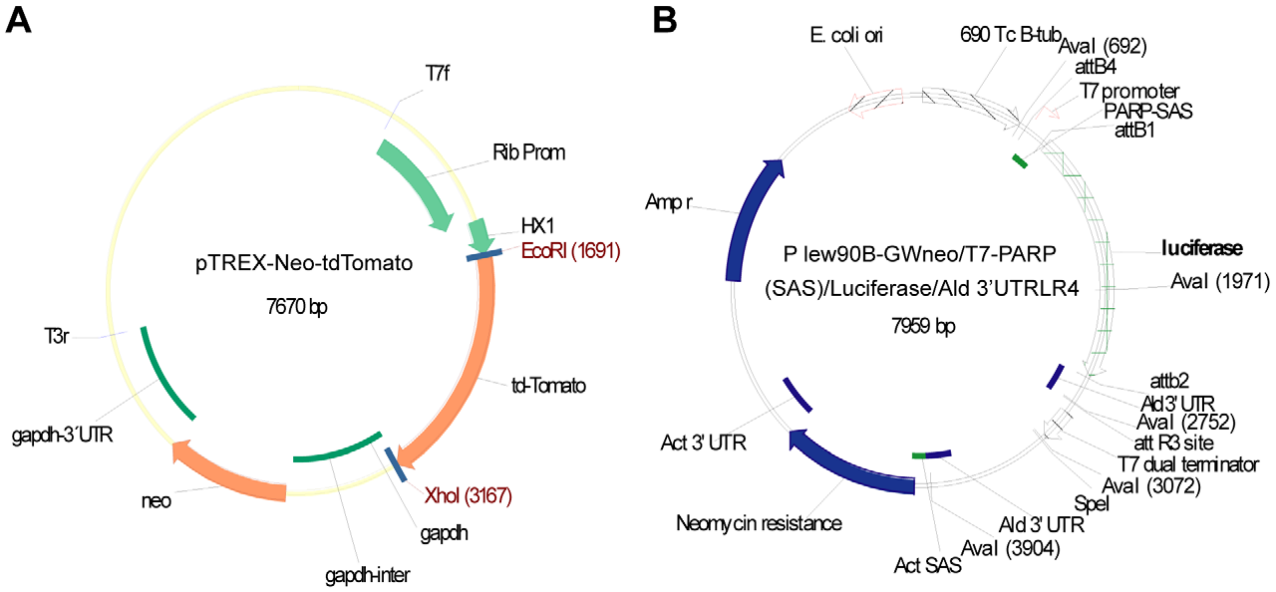
Plasmids used in the generation of fluorescent and bioluminescent *T. cruzi*. Schematic representation of (A) the pTrex-Neo-tdTomato plasmid, and (B) the pLew90β-GW/T7/PARP SAS/luciferase/Aldolase 3′UTR plasmid used for generation of the *T. cruzi* reporter lines.

To generate *T. cruzi* parasites expressing tandem dimeric tomato red fluorescent protein [25] we constructed a plasmid using the expression vector pTrex as a backbone [26]. The 1464 bp tdTomato gene was generated by PCR amplification using the primer set 5′-AGAATTCATGGCGCCTAGGGTGAGC-3′ (forward) and 5′-TACGTCGACTTAGAGCTCGATATCGACG-3′ (reverse), and the pCTR2t vector as the template [27]. The forward primer has an EcoRI site and the reverse primer has a SalI site. This PCR fragment was digested and cloned downstream of the rRNA gene promoter and the HX1 fraction into EcoRI and SalI sites in the multi-cloning site of the pTREX plasmid, generating the pTREX-tdTomato plasmid (Figure 1A).

Transgenic parasites were generated as previously described [28]. A total of 1×10^7^ early-log epimastigotes were centrifuged at 1,620 g for 15 min and suspended in 100 µl Human T Cell Nucleofector™ Solution (Lonza, Cologne) at room temperature. The resuspended parasites were then mixed with 10 µg DNA in a total volume of 10 µl and electroporated using the program “U-33” in an AMAXA Nucleofector Device (Lonza). The electroporated parasites were then cultured in 25 cm^2^ culture flasks (Corning Incorporated, Lowell, MA) with 10 ml LDNT medium and 300 µg/ml G418 was added at 24 h post-transfection. Parasite cultures are monitored by flow cytometry frequently for loss of fluorescence, and when necessary, sortedto remove non-fluorescent contaminants using a MoFlo cytometer (DakoCytomation, Carpinteria, CA) with an Enterprise 631 laser tuned to 488 nm for excitation and an emission filter with a band pass of 570/40 nm. Multiple strains of *T. cruzi* (CL, Tulahuen, Brazil, Colombiana and TCC) expressing the tdTomato protein were generated and the CL tdTomato strain used in the experiment shown here unless otherwise noted.

### Generation of bioluminescent *Trypanosoma cruzi*

To generate bioluminescent *T. cruzi* parasites, we constructed a plasmid based on Multi-Gateway technology (Invitrogen, Carlsbad, CA). The vector pLew90β was modified to include the Gateway cloning cassette by first inserting a new 62 bp multiple cloning site ( CCCGAG(AvaI)ACCGGTGTTAACGGGCC CTTCGAAGGTACCGAGCTCTTAATTAAGATATC(SpeI)ACTAGT ) into pLew90β at the Ava I and Spe I sites downstream of the Tc Beta Tubulin gene. The Multi-Gateway cloning cassette was digested from the pDEST-R4R3 (Invitrogen, Carlsbad, CA) plasmid using Pci I. A Klenow reaction was used to fill in the Pci I overhang. The fragment was then digested with BsaHI and cloned into pLew90β at HpaI/BstBI sites in the new multiple cloning site to create the pLew90β-GW-T7 destination plasmid. The combined T7 promoter and PARP splice acceptor site for the luciferase gene was cloned into the pDONR-P4P1R plasmid using a synthetic DNA construct in the pPCR-Script vector (GeneArt, Burlingame, CA). This fragment was digested from the pPCR-script vector with KpnI and Hind III (flanking sites underlined) then recombined into the pDONR-P4P1R (Invitrogen, Carlsbad, CA) plasmid utilizing the Gateway (Invitrogen, Carlsbad, CA) Att recombination sites (bold italics; GGTACC ***GGGGACAACTTTGTATAGAAAAGTTG***GGAGCTCGTAATACGACTCACTATAGGGCGAATTGGATCCTGCACGCGCCTTCGAGTTTTTTTTCCTTTTCCCCATTTTTTTCAACTTGAAGACTTCAATTACACCAAAAAGTAAAATTCACAAG***CAAGTTTGTACAAAAAAGCAGTCCCC*** AAGCTT ) was digested. The luciferase gene was amplified as an 1.8kb PCR product from the pPT7:O^tet^/Luc vector (a gift from John E. Donelson, University of Iowa, Iowa City, IA). The primers used were flanked by the appropriate Gateway Att recombination sites (bold italics) and were as follows: luciferase sense primer; ***GGGACAAGTTTGTACAAAAAAGCAGGCTCAACC***ATGGfoAAGACGCCAAAAACATAAAGAA, luciferase antisense primer; ***GGGGACCACTTTGTACAAGAAAGCTGGGTC***TTACACGGCGACTTTTCC GCCCTT CTTGG. The luciferase PCR fragment was then cloned into the pDONR-221 vector (Invitrogen, Carlsbad, CA) utilizing the Gateway Att recombination sites. A third vector was generated with the 3′UTR for the luciferase gene by amplifying the *T. cruzi* Aldolase 3′UTR sequence from the original pLew90β plasmid with flanking Gateway Att recombination sites (bold italics): Aldolase 3′UTR sense primer, ***GGGGACAGCTTTCTTGTACAAAGTGG***GGTCTTAAGGATCCTGCCCATT, Aldolase 3′UTR antisense primer, ***GGGGACAACTTTGTATAATAAAGTTGT***GCCCGGGCTCGAATCCC CCC. The Aldolase 3′UTR fragment was then cloned into the pDONR-P2RP3 vector (Invitrogen, Carlsbad, CA) utilizing the Gateway Att recombination sites. Finally, the pLew90β-GW-T7 (Destination) plasmid, pDONR-P4P1R/T7/PARP SAS, pDONR-221/luciferase, and pDONR-P2RP3/Aldolase 3′UTR plasmids were combined as per the Multi-Gateway protocol (Invitrogen Carlsbad, CA) to yield a final plasmid construct of pLew90β-GW/T7/PARP SAS/luciferase/Aldolase 3′UTR (Figure 1B). This vector was transfected into *T. cruzi* epimastigotes using the same protocol as described above for the tdTomato vector.

### Epimastigote growth inhibition assay

tdTomato epimastigotes (1×10^4^ parasites/well) were plated in 96 well Costar black plates (Corning Incorporated, Corning, NY) with or without drug and the change in fluorescence intensity was measured as surrogate of growth using a fluorescence plate reader (SpectraMax M2, Molecular Devices, Sunnyvale, CA) daily for 4 days. Benznidazole (Radanil®, Roche, Rio de Janeiro) tablets (100 mg) were pulverized, dissolved in nanopure (Milli-Q) water, then filter sterilized and used as the reference trypanocidal drug (positive control). EXO2 derivatives [29]–[32] (EXO2-04, EXO2-12, EXO2-17 and EXO2-36) were kindly provided by Jose C. Aponte and Gerald B. Hammond (University of Louisville, Louisville, KY) and were dissolved in DMSO, with a final concentration containing less than 0.1% DMSO per well. The 50% inhibitory concentration (IC_50_) values were determined by linear regression analysis on day 3 of culture.

### Amastigote growth inhibition assay

Vero cells were exposed to 2000 rad of gamma radiation for ten minutes [33] and 1.7×10^4^ cells were plated in 96-well plates overnight at 37°C/5% CO_2_. Vero cells were exposed to trypomastigotes of the CL tdTomato strain of *T. cruzi* for approximately 5 h at a multiplicity of infection (MOI) of 10. After infection, cell cultures were washed to remove non-internalized parasites and fresh media with or without benznidazole (as the reference trypanocidal drug) or test compounds were added. The change in fluorescence intensity was determined as a measurement of growth over 3–4 days of culture. The 50% inhibitory concentration (IC_50_) values were determined by linear regression analysis at day 3.

### *In vivo* drug testing of anti-*T. cruzi* compounds

Balb/c mice were purchased from the National Cancer Institute (Frederick, MD) and maintained in the University of Georgia animal facility in microisolator cages under specific pathogen-free conditions. For the short term *in vivo* assay mice were infected in each pad of both hind feet with either 2.5×10^5^ of *T. cruzi* trypomastigotes (CL strain) expressing the tdTomato protein or with 1×10^5^ trypomastigotes expressing luciferase. Mice were then submitted to benznidazole (BZ), ENH-5 and NTLA-1 (nitrotriazole derivatives; the gift of Maria Papadopulou, Northwestern Medical Center), Posaconazole, an antifungal triazole derivative (POS) [34], EXO2-04, or BIS767 (a bisphosphonate; the gift of Melina Galizzi and Roberto Docampo), treatment from day 6 to day 11 (tdTomato parasites) or from day 4 to day 10 (luciferase parasites) post infection. An untreated control, as well as a naïve non-infected group of mice was also monitored in each experiment. BZ was prepared by pulverization of one tablet containing 100mg of the active principle, followed by suspension in distilled water. BZ was administered orally, with daily doses of 100 mg/kg body weight. ENH-5 (20mg/kg/day) and NTLA-1 (2mg/kg/day) were suspended in PBS and daily injected intraperitoneally (i.p.) into mice while EXO2-04 (20mg/kg/day) was suspended in 1% DMSO and administered to the mice i.p. POS was dissolved in an aqueous solution of 2% methylcellulose and 0.5% Tween 80 and administered orally, with daily doses of 20 mg/kg body weight. BIS767 was suspended in PBS and daily injected subcutaneously into mice. The fluorescence and bioluminescent intensity (photons/cm^2^/sec) was measured before and after treatment as a surrogate of parasite load at the site of infection.

For the long term *in vivo* assay mice were infected i.p. with 1000 trypomastigotes of CL strain of *T. cruzi* and sacrificed by CO_2_ inhalation at different time points post infection. Infected mice were divided into the following groups: mice infected without specific treatment (untreated); mice treated orally with BZ, with daily doses of 100 mg/kg body weight for 40 days (15 to 55 dpi) (BZ40), mice treated with daily i.p doses of 2mg/kg/day of NTLA-1 for 50 days (15–65 dpi), mice treated orally with POS for 40 days (15–55 dpi) with daily doses of 20mg/kg/day (POS) and mice treated subcutaneously with BIS767 for 30 days (15–45 dpi) with daily doses of 500µg/kg/day (BIS767).

### *In vivo* bioluminescent and fluorescent imaging

Prior to bioluminescent imaging, mice were anaesthetized with 1.5% isofluorane and then injected with 150 mg/kg body weight of substrate luciferin potassium salt (Caliper, Hopkinton, MA) dissolved in PBS was administered by a single i.p. injection. Mice were imaged in a bioluminescent imaging system (IVIS 100; Xenogen, Alameda, CA). Briefly, mice were placed into the camera chamber, where a controlled flow of 1.5% isofluorane in air was administered through a nose cone via a gas anesthesia system. The luciferin substrate was allowed to adequately disseminate in the mice [35] for 10 min before imaging. Mice were imaged in dorsal, position by capturing a grayscale body image overlaid by a pseudocolor image representing the spatial distribution of the detected photons. Images were collected with 5 min integration time. Data acquisition and analysis were performed by using the Living-Image software (Xenogen, Alameda, CA) where luminescence could be quantified as the sum of all detected photon counts per second within a chosen region of interest. For *in vivo* fluorescent imaging, footpads of mice subcutaneously infected with 2.5×10^5^ tdTomato parasites in 4 ul were imagined every other day using the Maestro2 *In Vivo* Imaging System (CRi, Woburn, MA) with the green filter set (acquisition settings: 560 to 750 in 10 nm steps; exposure time 88.18 ms and 2×2 binning). Collected images were unmixed and analyzed with the Maestro software v2.8.0A.

### Statistical analysis

Statistical analysis was performed by ANOVA and unpaired T test, using the GraphPad PRISM 3.0 software. Differences between two groups were considered significant at p<0.05.

## Results

### Stable tdTomato expression in *T. cruzi*

**Figure 2:**
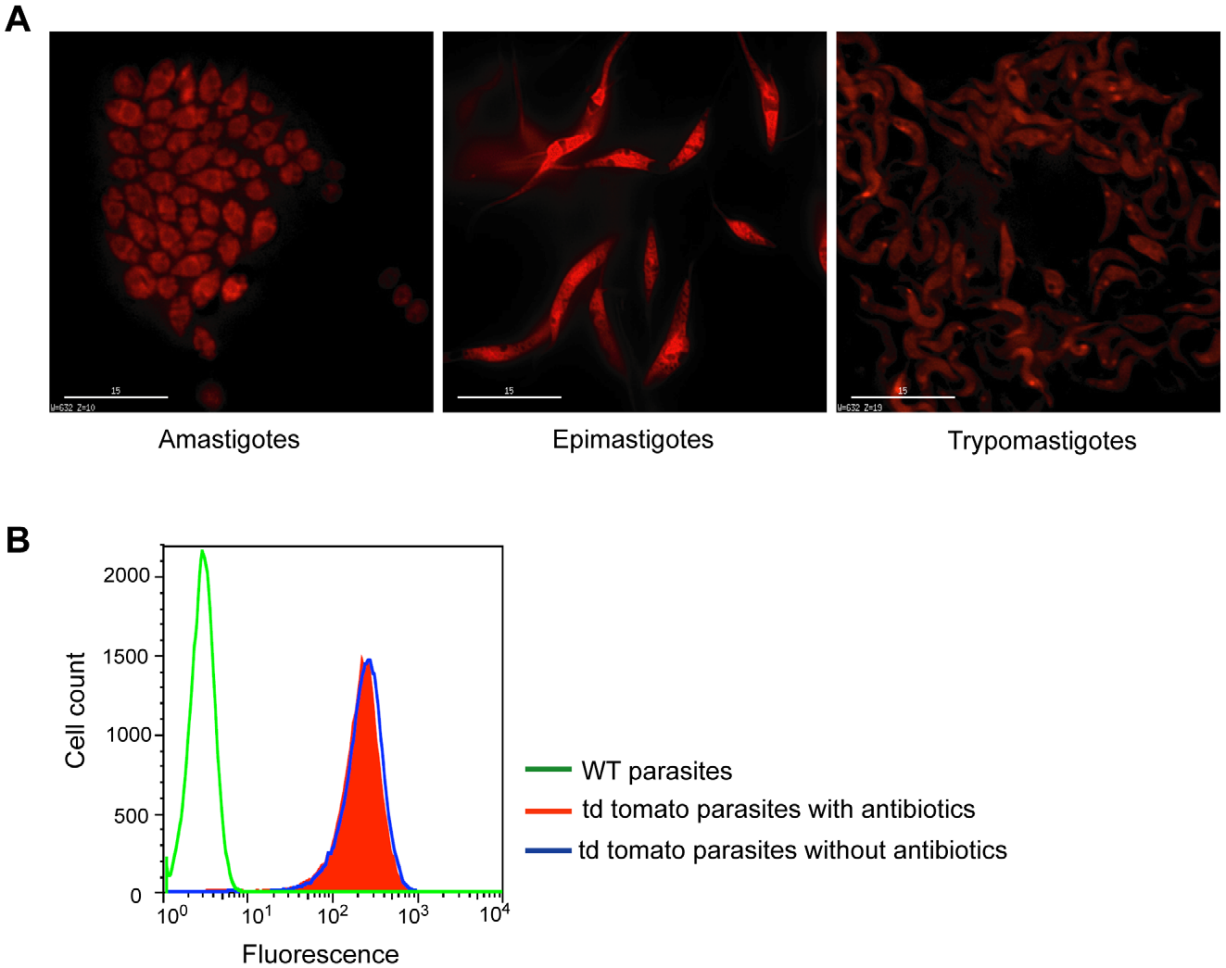
Fluorescence evaluation of tdTomato parasites. (A) Microscope image showing the tdTomato expressing parasites in all the life stages. (B) The fluorescence intensity in epimastigotes was assessed using a CyAn flow cytometer (DakoCytomation) and analyzed with FlowJo software (Tree Star). No decrease in fluorescence intensity was observed in parasites cultured for >5 months with (red) and without antibiotic (blue). The background fluorescence of WT parasites (green) can also be observed.

The tdTomato gene used in these studies was generated by genetically fusing two copies of the gene encoding the Tomato Red Fluorescent protein to create a tandem dimer (td) named tdTomato [25]. This construct possesses many desirable properties relative to previously studied fluorescent proteins, including a faster and more complete maturation and increased brightness [25], [35]. In addition, the tdTomato fluorescence signal can be detected at ≥620 nm (outside the range of of the bulk of animal tissue autofluorescence), which minimizes autofluorescence and greatly increases the penetration of fluorescence signals into the tissues [25], [36]. The expression vector pTrex-Neo [26], carrying the *T. cruzi* ribosomal promoter, has been shown to provide strong protein expression in *T. cruzi* and to enable the stable integration of exogenous genes into the ribosomal locus [26], [37]–[38]. Thus the gene encoding tdTomato was cloned into pTrex-Neo and the pTREX-Neo-tdTomato (Figure 1A) construct transfected into epimastigotes of various *T. cruzi* strains, which were then drug-selected as described in the Materials and Methods. Parasite fluorescence was monitored by microscopy and flow cytometry (Figure 2A–B). Transfectant parasites expressing the tomato protein showed a bright red fluorescence distributed throughout the cell in all life cycle stages (Figure 2A). Moreover, the fluorescence was stable in the absence of antibiotic pressure for >5 months (Figure 2B).

### *In vitro* drug screening assay

**Figure 3:**
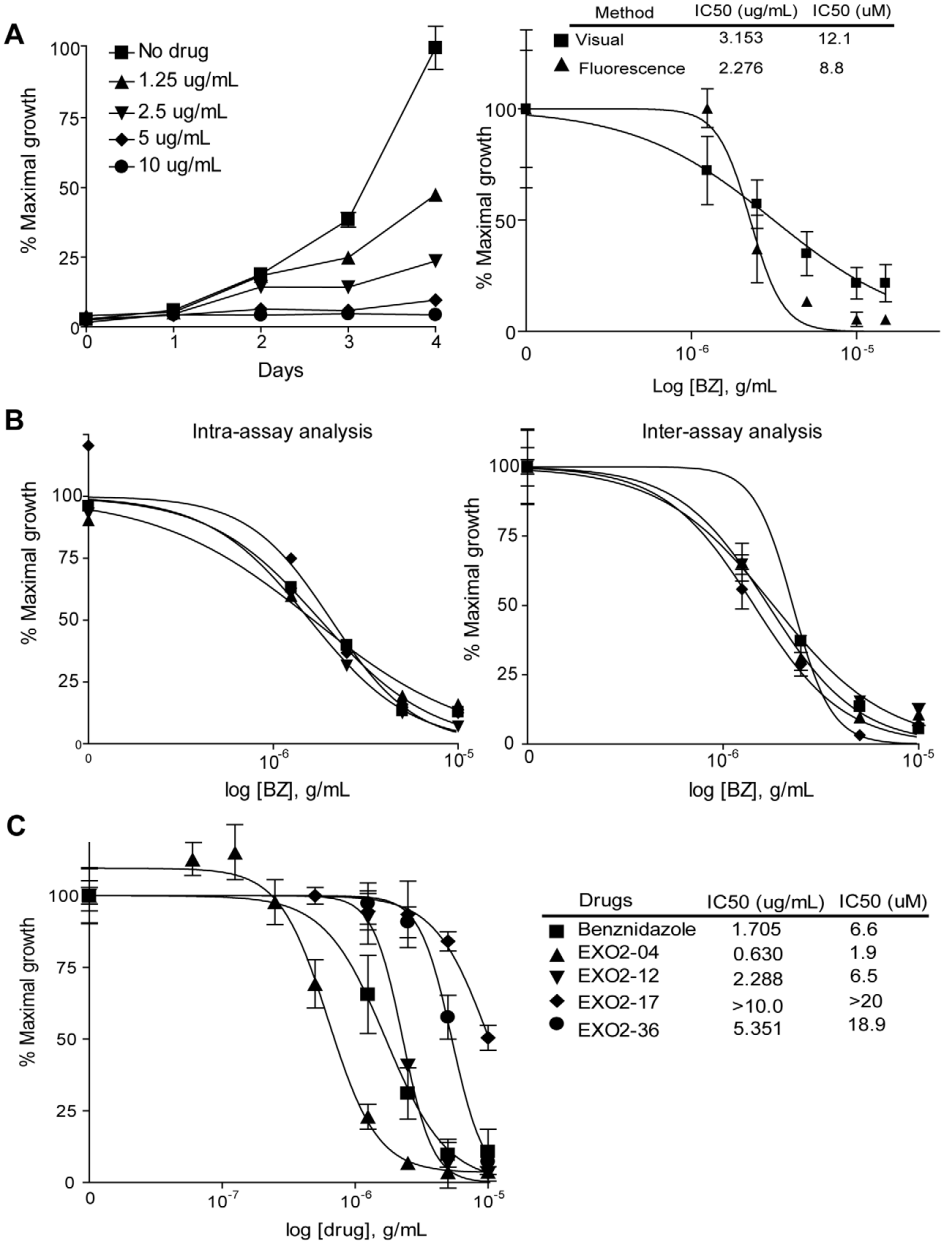
*In vitro* epimastigote growth assays using tdTomato parasites. (A) Epimastigotes growth over time in the presence of benznidazole at the indicated concentrations and comparison of measurement of drug inhibition of epimastigote growth by fluorescence and visual counting by hemacytometer. (B) Intra-assay analysis (left) showing the low variation among wells with the same drug concentration (n = 4). Inter-assay analysis (right) showing the low variation among IC_50_ curves from individual assays. (C) IC_50_ calculation in response to benznidazole and the EXO2 derivatives activity against epimastigotes after 3 days of treatment/culture.

Extracellular epimastigotes are easily obtained and thus are often used as a first step in the screening of new potential anti-*T. cruzi* drugs *in vitro* [39]. To address whether fluorescent tdTomato parasites were useful to screen for such compounds, we plated tdTomato epimastigotes with or without the proven anti-*T. cruzi* compound benznidazole (BZ) and the fluorescence intensity as a representative of parasite growth was measured daily. A dose dependent decrease in parasite growth was evident within 2 days of addition of BZ to cultures and was clearly evident throughout the remainder of the 4 day assay period (Figure 3A). The IC_50_ calculated for benznidazole is similar to that of other methods, including visual counting (Figure 3A), and as previously reported in the literature for several *T. cruzi* strains [40]–[41]. This method showed low intra-assay variation and a very good consistency by the inter-assay analysis (Figures 3B). The analysis of the test EXO2 compounds (EXO2-04, EXO2-12, EXO2-17 and EXO2-36) revealed one with IC_50_ below than that of BZ (Figure 3C).

**Figure 4:**
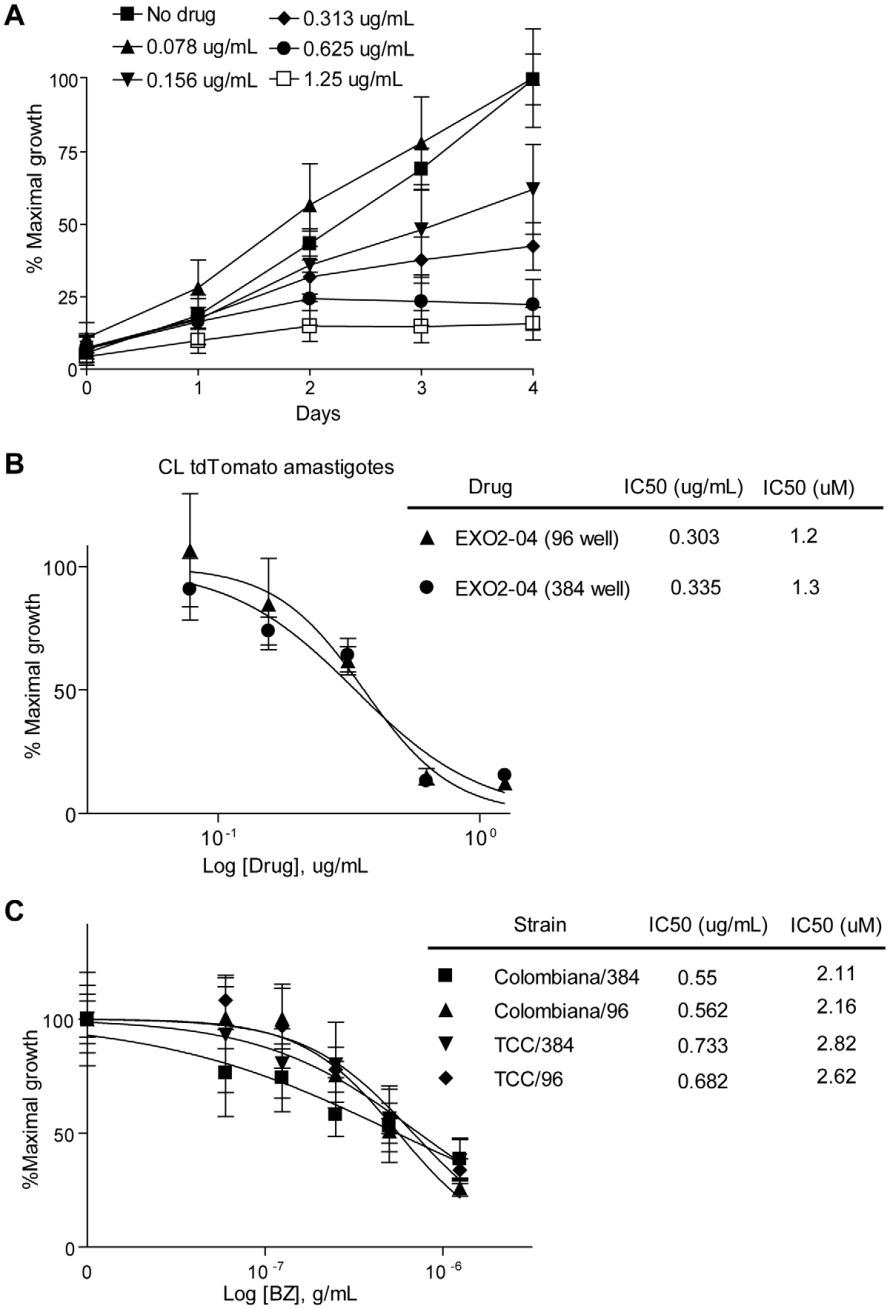
*In vitro* amastigote growth assays using tdTomato parasites. (A) Amastigotes growth in Vero cells grown in 96 well plates over time in the presence of benznidazole (n = 8). (B) Comparison of IC50 calculations in response to EXO2-04 in 96 and 384 well plates at 3 days of treatment (n = 4). (C) Amastigote growth assay in 96 or 384 well plates using the Colombiana and TCC strain of *T. cruzi* expressing tdTomato fluorescent protein at 3 days of treatment (n = 8).

The ability of compounds to inhibit the intracellular growth of *T. cruzi* amastigotes is a more rigorous and relevant test of anti-*T. cruzi* activity, as it is applied to a stage which is the predominant form in mammals and because the killing assay requires that drug also cross the host cell membrane. Amastigote growth assays in Vero cells worked similarly to epimastigotes assays, with parasite fluorescence increasing over the 4 day culture period. BZ exhibited a dose-dependent inhibitory effect on parasite growth (Figure 4A). Furthermore, this assay produced comparable results in 96 or in 384 well plates (Figure 4B–C), demonstrating the potential utility for the testing of large compound libraries. Lastly, the expression of tdTomato in different parasite strains which may differ in susceptibility to various drugs provides an easy method to confirm the susceptibility of multiple parasite strains to potential anti-*T.cruzi* compounds (Figure 4C).

### Use of tdTomato- and luciferase-expressing *T. cruzi* for determination of drug efficacy *in vivo*

We next explored the utility of *T. cruzi* lines expressing firefly luciferase or tdTomato for assessing the activity of compounds on the *in vivo* growth and survival of *T. cruzi*. In order to optimally visualize parasite development as well as to simulate a normal route of infection, parasites were delivered subcutaneously into the footpad of mice. Mice were then left untreated or were treated with various compounds starting at 6 days post-infection for those infected with tdTomato parasites or 4 days post infection for mice infected with bioluminescent parasites. These time points were chosen as they allowed sufficient time for establishment of the infection and easy visualization using the respective imaging technique. In addition to the proven compound BZ, a number of other compounds were tested with either assay, including EXO2 which had shown excellent IC_50_ in the *in vitro* assay (Figures 3 and 4), NTLA-1 and ENH-5, two nitrotriazole derivatives also previously demonstrated to have high *in vitro* activity against *T. cruzi* amastigote growth (M. Papadopoulou personal communication), a bisphosphonate (BIS767) with *in vitro* anti-*T. cruzi* activity (Galizzi and Docampo personal communication) and the antifungal posaconazole (POS), which has been reported to have *in vivo* trypanocidal activity [22], [42]. In all cases the compounds were delivered systematically, by oral gavage in the case of BZ and POS and by i.p. injection in the cases of the EXO2-04, NTLA-1 and ENH-5 and by a subcutaneous injection in the ventral abdomen in the case of BIS767.

**Figure 5:**
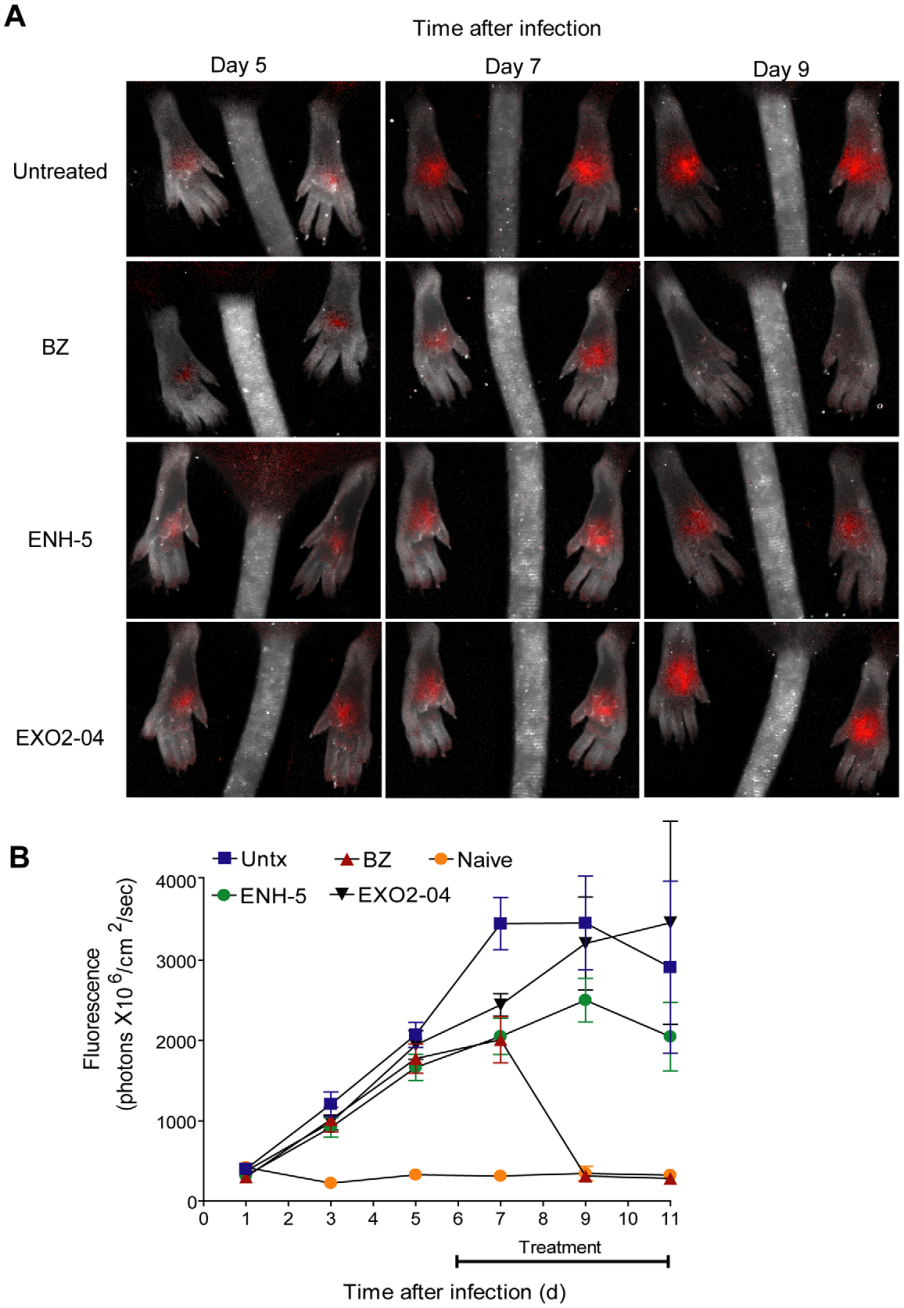
Fluorescent *T. cruzi*-tdTomato expressing parasites imaged post-treatment. Mice (10 per group) were infected in the hind foot pads with 2.5×10^5^ *T. cruzi* tdTomato trypomastigotes and the images were taken every two days from day 1 to 11 post infection. (A) Images from days 5, 7 and 9 post infection. (B) Quantification of the fluorescent signal from mice in panel A at all imaging points.

**Figure 6:**
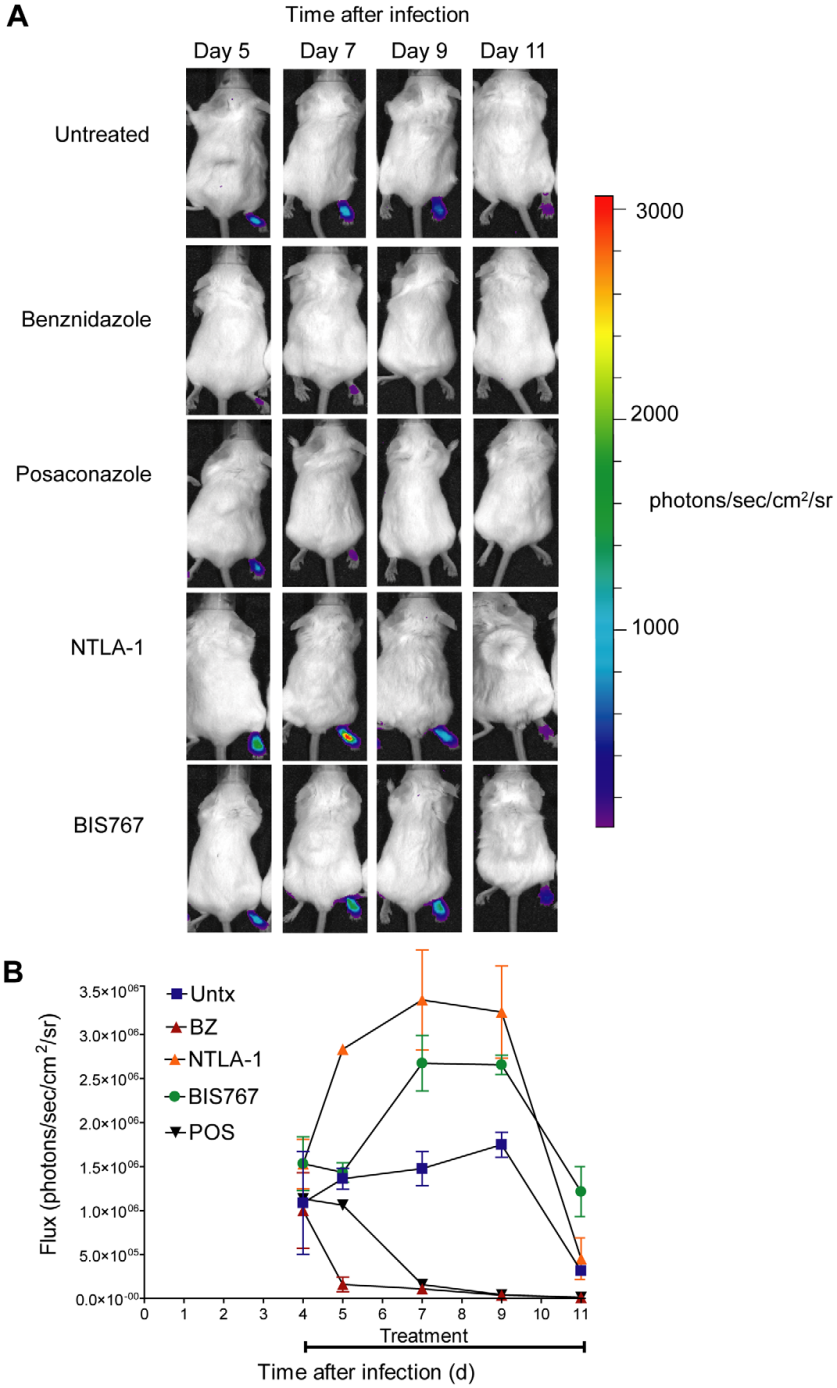
Luminescent *T. cruzi* imaged at various times post-treatment. (A) Mice (10 per group) were infected in the footpad with 1×10^5^ *T. cruzi* bioluminescent trypomastigotes. For all images shown the color scale ranges from blue (with a minimum set at 60 photons/s/cm^2^/sr) to red (maximum of 3000 photons/s/cm2/sr). (B) Quantification of luminescent signal from mice in panel A.

Both the tdTomato and the luc-based assays revealed a rapid in vivo parasite clearance activity for BZ and POS, with dramatic control of parasite load within 1 day with BZ and slightly longer in the case of POS (Figures 5 and 6). In contrast, none of the other test compounds exhibited significant effects on *in vivo* parasite growth, despite their previously demonstrated *in vitro* anti-*T. cruzi* activity.

### Suppression of parasitemia is not indicative of parasite clearance

**Figure 7:**
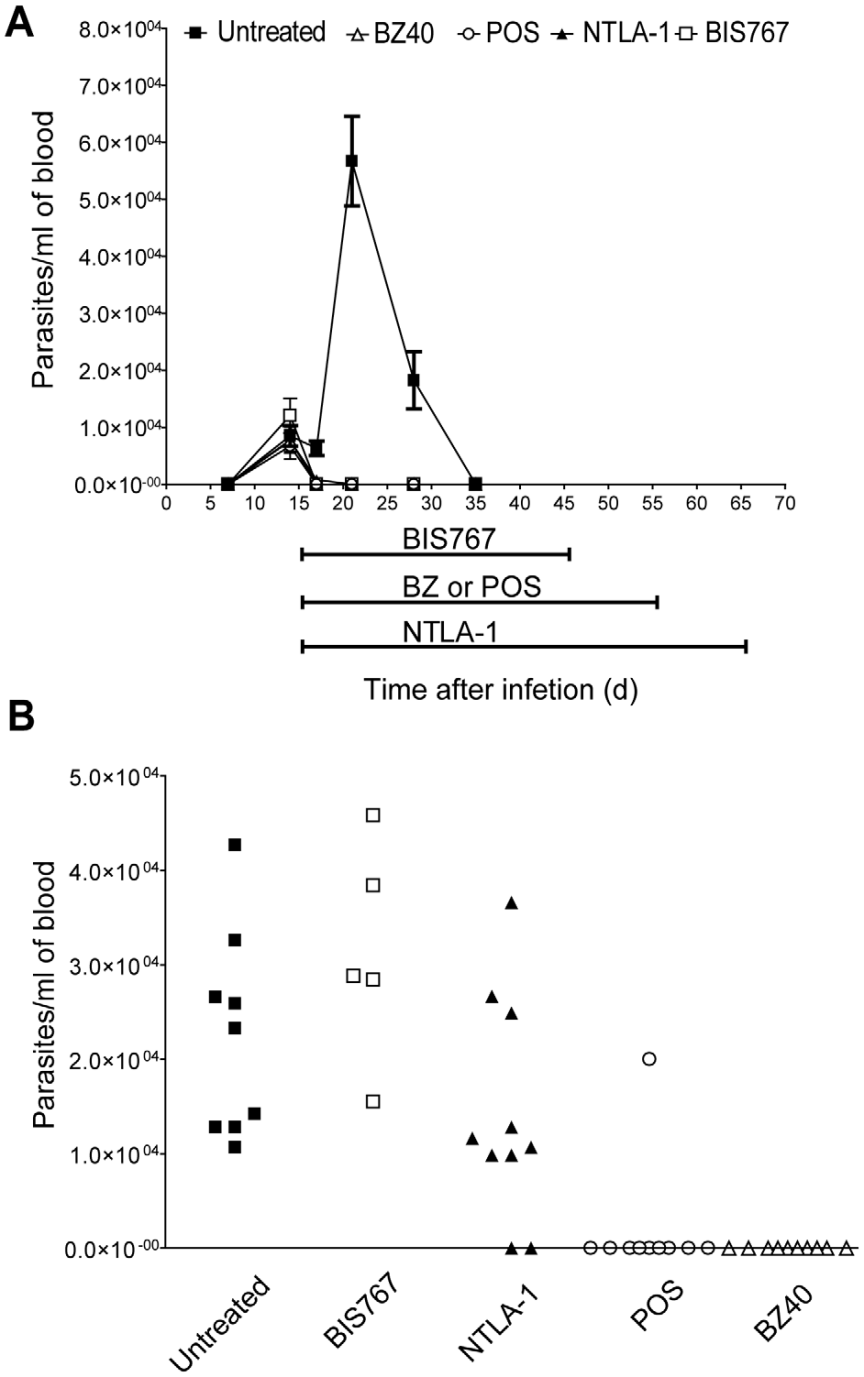
Rapid suppression of parasitemia following drug-treatment is a poor indicator of drug efficacy and parasitological cure. (A) Evolution of parasitemia after infection with 1×10^3^ CL strain of *T. cruzi* on day 0 in untreated (▪), BZ-40 (▵), POS (○), NTLA-1 (▴), or BIS767 (□) treated mice. “BIS767, BZ-40, POS and NTLA-1” bars below x axis indicate period of treatments. (B) Parasitemias in untreated or treated mice at 120dpi, after administration of the immunosuppressant cyclophosphamide (cy) (days 105, 108, 111, 113 and 117).

In previous work, we showed that BZ treatment in the acute or chronic phase of the infection can provide cure of mice infected with *T. cruzi* [43]. We sought to explore whether the same compounds used in the *in vivo* short term assays were effective to cure mice infected with *T. cruzi*. To address this question, C57BL/6 mice were infected with the wild-type CL strain of *T. cruzi* and treated with either BZ, POS, NTLA-1 or BIS767 or left untreated. All mice exhibited detectable parasitemias by day 14 post-infection; in untreated mice this acute phase parasitemia peaked at 21 dpi and became undetectable by approximately 35 dpi (Figure 7A). All of the compounds evaluated in this study suppressed parasitemia, which became undetectable in all cases by 21 dpi (Figure 7A). However, only the mice treated for 40 days with BZ, the majority of POS-treated (90%) and a small fraction of NLTA-1-treated mice (20%) were able to clear the infection and cure, as by the failure to detect parasitemias after cyclophosphamide (cy) immunosuppression (Figure 7B). These results demonstrate that suppression of parasitemia soon after drug-treatment initiation Figure 7A) is a poor indicator of drug efficacy and the potential for a compound to achieve parasitological cure over a long-term course of treatment. Moreover, the results of the long-term-treatment assay and the short-term *in vitro* screens using measurement of parasite growth in the foot-pad following the injection of luminescent or fluorescent *T. cruzi* (Figures 5 and 6) are perfectly concordant, suggesting that the short-term in vivo assay is strong predictor of in vivo drug efficacy and clearly superior to measuring suppression of parasitemia.

## Discussion

There is a clear need for new compounds to treat *T. cruzi* infection at all stages of the infection/disease [3], [6], [44]–[45]. One crucial limitation in the identification of such compounds is the lack of adequate and efficient in vitro and in vitro tests of drug efficacy. Various colorimetric and fluorometric assays have been developed to screen for anti-*T. cruzi* compounds [7]–[12] which are more accurate and objective than the microscopic visual counting. More recently, an efficient method to quantify *T. cruzi* parasites in drug screening assays using genetically engineered parasites that express the *Escherichia coli* β-galactosidase gene, *lacZ* has been described [15]. Although widely and effectively used and even scaled to a 384 well format (Ana Rodriguez, personal communication), this method depends on a single endpoint reading following the addition of detergent and a convertible substrate. *T. cruzi* lines expressing alternative reporters such as luciferase [46], fluorescent proteins [37], [47] have also been produced, but the use of these parasites lines for *in vitro* and *in vivo* screening of potential anti-*T. cruzi* compounds has not been reported.

Herein, we report the generation of stable *T. cruzi* lines constitutively expressing the tandem tomato fluorescence protein (tdTomato) or the firefly luciferase protein and the use of these lines to screen for anti-*T.* cruzi compounds in vitro and to confirm their activity in vivo. tdTomato-expressing parasites showed strong fluorescence in all life stages including when the parasites were maintained in the absence of antibiotic selection. Among the advantages of the use of these fluorescent parasites for in vitro assays is the elimination of the need for fixation or cell permeabilization, and detection of fluorescence with minimal handling using a fluorimeter, microscope-based screener or other imaging system. The use of fluorescent parasites also allows for the continuous measurement of parasite replication during the experiment, making possible the analysis of the parasite growth and/or the initiation and cessation of growth inhibition by drugs over time. The fluorescence-based growth inhibition assays of both epimastigotes and intracellular amastigotes had high intra- and inter-assay consistency and could be easily scaled to at least a 384 well format, allowing for the development of high throughput screening of large compound libraries. The assays also work well in multiple *T. cruzi* strains; to date we have produced TdTomato expressing parasites from all 4 *T. cruzi* lines in which we have attempted.

While the in vitro drug screening assay is a significant improvement in methodologies for testing of anti-*T. cruzi* compounds, the use of *T. cruzi* lines expressing luciferase and tdTomato for in vivo compound screening bridges a more significant technical gap and thus represent a much bigger step forward. The current standard for the in vivo testing of anti-*T. cruzi* compounds is the measurement of the suppression of parasitemia in the acute phase of the infection or the protection from acute-phase death, neither of which assess parasitological cure [18]–[22]. We have recently developed an in vivo treatment protocol that critically evaluates parasitological cure by using suppression of immune responses following drug treatment, a procedure that releases constraints on parasite growth and thus reveals drugs and treatment regimens that fail to completely cure the infection [43]. In this study, we have used this immunosuppression technique as the gold standard for drug-induced cure. However, the considerable drawback of this protocol is the long course of the experiment, including ∼40 days for compound treatment and a minimum of 75–80 days for the entire experiment. Herein we demonstrate that a short-term treatment protocol, involving 5–6 days of treatment and a total of 11–12 days for the full experiment, that assesses the suppression of parasite replication (although not full clearance) at subcutaneous sites. The assay is not only quick, but is also quite easy, requiring short-term compound dosing and occasional animal imaging. Most importantly, for the limited set of compounds tested to date, this short-term assay using either bioluminescent or fluorescent parasites gives results that are nearly identical to those obtained using the long-term treatment protocol followed by immunosuppression; In both assays BZ is most highly effective compound of those tested and POS is slightly less effective.

A particularly notable point from the results of these different screening assays is that multiple compounds that strongly suppress epimastigote and amastigote growth in vitro, and furthermore, rapidly and significantly suppress the level of parasites in the blood of acutely infected mice, fail to cure mice in the long term treatment assays or to suppress parasite replication in the tissues in the short-term assay. Although more compounds will have to be compared using both the short- and long-term in vivo protocols used herein, the results to date are highly encouraging that the short-term assay measures a drug effect that is more like what is required for parasitogical cure and thus is a better screen for in vivo efficacy than the acute suppression of parasitemia. Certainly, these two assays are likely measuring very different parameters, with suppression of parasitemia highlighting drugs that may have anti-parasite effects largely or solely on extracellular parasites. It is especially interesting that compounds that clearly suppress intracellular parasite replication in vitro fail to control parasite growth in the short term in vivo assays, despite the fact that the parasites are largely intracellular during these short-term in vivo tests (as they would have to be if they are increasing in number as indicated by the increase in fluorescent signal over time).

Although the short-term in vivo assay is quick and straightforward, there may also be room for additional improvements. A common limitation of these assays is the inability of fluorescent or bioluminescent signals to easily penetrate animal tissues, including fur. For this reason, we monitored parasite growth in subcutaneous hairless sites (footpads (shown here)) and ears (data not shown), although luciferase-expressing parasites can also be visualized throughout the body when infections are given at higher doses and/or at later time points in the infection (data not shown and [46]). The sensitivity of detection of the luciferase-expressing parasites is greater than that of the tdTomato parasites. However this advantage is surpassed by the fact that visualization of the tdTomato parasites does not require the repeated injection of the luciferase substrate nor a requirement to control for the time post-substrate injection that the imaging is done. Given that the beneficial effects of both BZ and POS are obvious within a day or 2 post-administration, it is also possible that the identification of drugs equivalent in efficacy to BZ may require only a single administration of the compound and only one or two imagining time points. These modifications would make it possible to screen 100's of compounds in vivo in very short order. Compounds that give a positive result could then be submitted to the longer-term treatment/immunosuppression assay.

The focusing of parasites largely at the site of infection was also evident in these experiments and contributed to the success of this technique for assessing drug treatments. The imaging of the luciferase or tdTomato parasites is not sufficiently sensitive to detect the movement of small number of parasites from the subcutaneous injection point to other distant sites. However it is clear that the vast majority of parasites remain at the injection site, where they infect cells and replicate. Interestingly, even in the absence of drug treatment, parasite load at the site of injection diminishes after ∼10 days post infection. Preliminary results suggest that this reduction is immune mediated, as it does not occur in mouse strains with defects in T cell responses, and suggest that whole animal imagining of parasite growth and dissemination might also be useful in the study of immune control of *T. cruzi* at tissue sites Padilla, et al, unpublished).

The tools described in this paper were dependable, facile and low cost and provide new methods for the rapid screening of anti-*T cruzi* compounds *in vitro* and *in vivo*. These methods should make feasible the broader and higher-throughput screening programs needed to identify potential new drugs for the treatment of *T. cruzi* infection.
